# Stigmasterol ameliorates obesity-associated insulin resistance by activating the PPARγ pathway and alleviating oxidative stress

**DOI:** 10.3389/fendo.2026.1888104

**Published:** 2026-07-28

**Authors:** Yanan Zhao, Fangfang Wang

**Affiliations:** 1 Qilu Medical University, Zibo, Shandong, China; 2 Yantaishan Hospital, Yantai, Shandong, China

**Keywords:** insulin resistance, network pharmacology, obesity, oxidative stress, PPARγ, stigmasterol

## Abstract

**Objective:**

This study aimed to investigate the effects of stigmasterol on obesity-related insulin resistance and to explore the underlying molecular mechanisms.

**Methods:**

Network pharmacology was used to screen the active components and key targets of Angelica sinensis that act on obesity and insulin resistance, and molecular docking was performed to assess the binding potential between stigmasterol and PPARγ. In cell experiments, an insulin-resistant 3T3-L1 adipocyte model was treated with different concentrations of stigmasterol and rosiglitazone. Lipid accumulation, oxidative stress indicators, and protein expression of the PPARγ pathway were measured. In animal experiments, high-fat diet-induced obese mice were administered stigmasterol and rosiglitazone, and serum metabolic parameters, histopathological changes, and hepatic oxidative stress and PPARγ pathway protein expression were measured.

**Results:**

Network pharmacology identified 90 overlapping targets, with PPARγ occupying a central position in the core network. Molecular docking indicated that stigmasterol showed good binding affinity for PPARγ. In insulin-resistant 3T3-L1 adipocytes, stigmasterol reduced intracellular TG and TC contents and lipid droplet accumulation, decreased ROS and MDA levels, increased the activities of CAT, GPx, and SOD, and upregulated the protein expression of PPARγ, the p-Akt/Akt ratio, and GLUT4 (p < 0.05). In obese mice, stigmasterol decreased the serum levels of fasting insulin, fasting blood glucose, HOMA-IR, TG, and TC, increased HOMA-ISI, reduced adipocyte size, alleviated hepatic steatosis, and lowered serum ALT and AST levels. Meanwhile, stigmasterol reduced hepatic ROS and MDA levels, restored antioxidant enzyme activities, and upregulated the protein expression of PPARγ, p-Akt/Akt, and GLUT4 in the liver (p < 0.05).

**Conclusion:**

Stigmasterol improved obesity-related glycolipid metabolic disorder and insulin resistance both *in vitro* and *in vivo*, and its mechanism involved activation of the PPARγ pathway and attenuation of oxidative stress.

## 1 Introduction

Rapid global urbanization and modernization have led to profound changes in lifestyle, including unhealthy diets, reduced physical activity, increased stress, and environmental exposures, which together have contributed to a sharp rise in the incidence of obesity and type 2 diabetes mellitus (T2DM). Insulin resistance is prevalent among obese individuals (1). It is characterized by impaired cellular responses to insulin in peripheral tissues such as the liver, skeletal muscle, and adipose tissue. This impairment is largely driven by mitochondrial reactive oxygen species (ROS)-mediated oxidative stress that disrupts insulin signaling pathways; therefore, oxidative stress plays a critical role in the pathogenesis of T2DM and its complications (2, 3). Accordingly, alleviating oxidative stress has been recognized as an effective strategy for ameliorating obesity-associated insulin resistance (4).

The peroxisome proliferator-activated receptors (PPARs) belong to the ligand-dependent nuclear receptor family and include three subtypes: PPAR-α, PPAR-β/δ, and PPAR-γ (5). PPAR-γ is highly expressed in adipose tissue and is regarded as a key regulator of lipid synthesis and metabolic homeostasis (6). Activated PPAR-γ enhances insulin sensitivity in peripheral tissues by regulating the transcription of downstream target genes (7). More recent studies have also revealed that PPAR-γ activation induces the expression of various antioxidant enzymes, thereby strengthening the capacity for ROS clearance and negatively regulating intracellular oxidative stress (8, 9). Thiazolidinediones (TZDs), which are classical full agonists of PPAR-γ, can effectively improve insulin resistance; however, their clinical application is largely limited by side effects such as weight gain, fluid retention, and increased cardiovascular risk (10). Therefore, the search for natural ligands that can gently modulate PPAR-γ activity while possessing a favorable safety profile has become an important direction in the field of metabolic regulation.

Angelica sinensis has long been used for metabolic disorders and accumulating evidence suggests its potential relevance to obesity and insulin resistance (11, 12). Among its bioactive constituents, stigmasterol (Sti) is a phytosterol with both anti-inflammatory and neuroprotective activities (13). Previous studies have reported that stigmasterol exerts neuroprotective, anticancer, antidiabetic, anti-osteoarthritis, and immunomodulatory effects (14). More importantly, recent findings have indicated that stigmasterol can regulate lipid metabolism and may influence the expression and activity of nuclear receptors such as PPARγ (15, 16). These biological properties suggest that stigmasterol has the potential to intervene in obesity-related metabolic disorders. However, whether stigmasterol can effectively alleviate obesity and the associated insulin resistance *in vivo* has not yet been reported, which represents a gap that remains to be filled.

Based on the above background, the effects of Sti on obesity-related insulin resistance and the underlying molecular mechanisms have not been systematically investigated. Moreover, although computational approaches such as network pharmacology and molecular docking can provide clues for identifying targets of natural products, experimental validation guided by these predictions remains limited. Therefore, the present study was designed to investigate the effects of Sti on obesity-associated insulin resistance and its relationship with the PPARγ/oxidative stress pathway, using a strategy that combines network pharmacology screening with both *in vitro* and *in vivo* experimental verification. This study will provide new experimental evidence for the metabolic regulatory functions of Sti and offer a theoretical basis for the identification of safe and effective phytosterol-based interventions for insulin resistance.

## 2 Methods

### 2.1 Collection of chemical constituents of Angelica sinensis and target prediction

The TCMSP database was used to retrieve all active components of Angelica sinensis under the criteria of oral bioavailability (OB) ≥ 30% and drug-likeness (DL) ≥ 0.18. The active components and their corresponding targets were screened and obtained. The BATMAN-TCM database was also used to retrieve active components and targets under the criteria of P ≤ 0.05 and score ≥ 20. The components and targets derived from both databases were merged. The collected targets were then calibrated against the UniProt database (https://www.uniprot.org/), after which non-human genes were removed and invalid duplicate targets were deleted to obtain standardized gene names.

### 2.2 Acquisition of disease-related targets

The GeneCards and OMIM databases were searched using the keyword “obesity” to retrieve obesity-related targets. All targets obtained from the two databases were integrated in Excel, and duplicate genes were removed. The resulting targets were then calibrated against the UniProt database to obtain the gene information for obesity-related targets. Similarly, the same databases were searched using the keyword “insulin resistance”, and the retrieved targets were integrated, deduplicated, and calibrated following the same procedure to obtain the gene information for insulin resistance-related targets. The targets shared by both conditions were taken as the disease targets.

### 2.3 Identification of overlapping targets

The drug targets of Angelica sinensis and the disease targets were pooled, and the overlapping targets were identified. A Venn diagram was then generated using the online platform (http://bioinfogp.cnb.csic.es/tools/venny/index.html).

### 2.4 Construction of the protein-protein interaction network of the overlapping targets

The overlapping gene targets were submitted to the STRING database to construct a protein-protein interaction (PPI) network. The species was set to “Homo sapiens” and the minimum required interaction score was set to 0.4 to ensure reliability; all other parameters were kept at the default settings. The resulting file was saved in TSV format and imported into Cytoscape for visualization. The CytoHubba plugin was further applied to evaluate the topological properties of the network and to identify the core targets through which Angelica sinensis acts on obesity and insulin resistance.

### 2.5 GO enrichment analysis and KEGG pathway analysis

The overlapping gene targets were submitted to the STRING database, with the gene identifier set to OFFICIAL_GENE_SYMBOL and the species set to Homo sapiens. The analysis function was used to annotate the roles of the target proteins in gene functions from the perspectives of Biological Process (BP), Cellular Component (CC), and Molecular Function (MF). To further clarify the involvement of these targets in signaling pathways, KEGG pathway enrichment analysis was performed. The top 10 enriched terms in BP, CC, and MF, together with the top 30 enriched KEGG pathways, were selected as the main functional categories and signaling pathways through which Angelica sinensis treats the disease, and were used to predict the underlying mechanisms.

### 2.6 Molecular docking

The three-dimensional (3D) structures of proteins(PPARγ, PDB ID: 4YT1) were obtained from the PDB database (https://www.rcsb.org/). PyMOL 2.3.0 was used to remove water molecules, unrelated protein chains, and the original ligands. The 3D structures of the bioactive components(Stigmasterol, PubChem CID: 5280794) were downloaded from the PubChem database (https://pubchem.ncbi.nlm.nih.gov/) and optimized using Chem3D with molecular force fields to obtain the most stable conformations with the lowest energy. Semi-flexible docking was performed using AutoDock Vina 1.2.0. The docking grid box was defined with the Grid module, centered at coordinates x = 21.562, y = 38.93, z = 25.455, with dimensions of 83.7 × 83.7 × 83.7 Å, and the Lamarckian genetic algorithm was adopted for the docking calculation. The binding free energies were calculated and the results were analyzed.

### 2.7 Cell culture and differentiation

3T3-L1 preadipocytes (Cell Bank of the Chinese Academy of Sciences, Shanghai, China) were cultured in DMEM supplemented with 10% newborn calf serum. When the cells reached 90% confluence, they were maintained for an additional 2 days(designated as day 0), the medium was replaced with differentiation induction medium (DMEM containing 10% FBS, 0.5 mmol/L 3-isobutyl-1-methylxanthine (IBMX, HY-12318, MCE, USA), 1 μmol/L dexamethasone (DEX, HY-14648, MCE, USA), and 10 μg/mL insulin (PB180432, Procell, China)) (17, 18). IBMX, dexamethasone, and insulin were added directly to the complete culture medium. After 2 days, IBMX and dexamethasone were withdrawn. On day 4, insulin was also removed. The cells were then maintained in DMEM supplemented with 10% FBS, with the medium refreshed every 2 days, until day 8, when mature adipocytes were obtained. Throughout the differentiation process, cell morphology was monitored under an inverted microscope. Undifferentiated cells appeared spindle-shaped or polygonal with small volume and irregular arrangement; after induction, the cells gradually rounded, increased in volume, and accumulated intracellular lipid droplets, indicating successful differentiation.

### 2.8 Cell viability assay

Cell viability was assessed using the Cell Counting Kit-8 (CCK-8; Dojindo, Japan). 3T3-L1 preadipocytes cells were seeded into 96-well plates at a density of 5000 cells per well and treated with stigmasterol (Merck, Germany) at concentrations of 0, 1.25, 2.5, 5, 10, 15, and 20 μM for 48 h. Subsequently, 10 μL of CCK-8 reagent was added to each well, and the plate was incubated for an additional 1.5 h in a 5% CO_2_ incubator. The absorbance of each well was measured at 450 nm using a microplate reader.

### 2.9 Detection of intracellular ROS

Mature adipocytes were divided into six groups: control group (equal volume of vehicle), model group (1 μmol/L dexamethasone for 48 h), rosiglitazone positive control group (20 μmol/L RSG + 1 μmol/L dexamethasone for 48 h), Sti low-dose group (2.5 μmol/L Sti + 1 μmol/L dexamethasone for 48 h), Sti medium-dose group (5 μmol/L Sti + 1 μmol/L dexamethasone for 48 h), and Sti high-dose group (10 μmol/L Sti + 1 μmol/L dexamethasone for 48 h). After treatment, the cells were loaded with DCFH-DA probe (10 μmol/L, 37 °C, 20 min), washed, and the intracellular ROS levels were measured. The ROS Assay Kit was purchased from Beyotime.

### 2.10 Animals

Four-week-old SPF male C57BL/6 mice (body weight 14–16 g) were purchased from Shandong Pengyue Laboratory Animal Technology Co., Ltd. After one week of adaptive feeding, the mice were randomly divided into six groups (n = 6 per group): control group (normal chow diet, daily intragastric administration of an equal volume of normal saline), model group (high-fat diet, daily intragastric administration of an equal volume of normal saline), rosiglitazone positive control group (high-fat diet, intragastric administration of rosiglitazone 20 mg/kg/day), Sti low-dose group (high-fat diet, intragastric administration of Sti 200 mg/kg/day), Sti medium-dose group (high-fat diet, intragastric administration of Sti 400 mg/kg/day), and Sti high-dose group (high-fat diet, intragastric administration of Sti 600 mg/kg/day). The normal chow diet consisted of 20% protein, 70% carbohydrate, and 10% fat, and the high-fat diet consisted of 20% protein, 20% carbohydrate, and 60% fat. After 12 weeks of continuous intervention, all mice were fasted for 12 h and then sacrificed the following day by intraperitoneal injection of an overdose of sodium pentobarbital (150 mg/kg) under anesthesia. Blood and tissue samples were collected for subsequent analyses. The experimental protocol was approved by the Animal Ethics Committee of Qilu Medical University (approval number: YXLL2026D090).

### 2.11 Oil Red O staining

Lipid deposition was detected by Oil Red O staining (Beyotime, China). For liver tissues, liver tissues were rinsed with PBS, fixed with 4% paraformaldehyde, embedded in OCT compound, and sectioned using a cryostat. For staining, frozen sections were prepared, rinsed with PBS, and then stained with Oil Red O solution for 10 min. Subsequently, the sections were differentiated in 80% propylene glycol for 1 min, counterstained with hematoxylin, rinsed with water, mounted, and observed under a microscopic imaging system. For cells, cells cultured in six-well plates at a density of 1 × 10^5^ cells/cm² were collected, rinsed with PBS, fixed with 10% formaldehyde for 10 min, stained with Oil Red O solution(prepared by mixing Oil Red O stock solution with diluent at a ratio of 3:2) for 30 min, differentiated in 80% propylene glycol for 1 min, stained with hematoxylin for 1 min, rinsed with water, and observed and photographed under a microscope.

### 2.12 Biochemical assays

The Mouse Insulin Enzyme-Linked Immunosorbent Assay Kit (PI602), Amplex Red Triglyceride Assay Kit (S0219S), Amplex Red Cholesterol and Cholesteryl Ester Assay Kit (S0211S), Superoxide Assay Kit (S0060), Cellular Glutathione Peroxidase Assay Kit with NADPH (S0056), Catalase Assay Kit (S0051), and Lipid Peroxidation MDA Assay Kit (S0131S) were all purchased from Beyotime Biotechnology (Shanghai, China). The Amplex Red Alanine Aminotransferase Activity Assay Kit (P2711S) and Amplex Red Aspartate Aminotransferase Activity Assay Kit (P2715S) were also obtained from Beyotime Biotechnology. All assays were performed according to the manufacturers’ instructions.

### 2.13 Western blot analysis

Cells were washed with PBS and lysed thoroughly with RIPA lysis buffer (Merck, Germany). For tissue samples, an appropriate amount was minced, mixed with pre-cooled RIPA lysis buffer, and homogenized on ice. Both cell lysates and tissue homogenates were centrifuged at 12,000 g for 10 min at 4 °C. The supernatant was collected, and protein concentrations were determined using the BCA Protein Quantification Kit (Beyotime, China). Equal amounts of protein were loaded and separated on 10% SDS-PAGE gels. The proteins were then transferred onto PVDF membranes at a constant current of 250 mA for 2 h. The membranes were blocked with 5% non-fat milk at room temperature for 1 h and subsequently incubated with primary antibodies at 4 °C overnight. The following day, the membranes were washed three times with TBST (5 min each) and incubated with the secondary antibody (Goat Anti-Rabbit IgG (H+L), LF102, 1:10,000) at room temperature for 1 h. Protein bands were visualized using ECL chemiluminescence reagent (Epizyme Biomedical, China) and analyzed with ImageJ software for grayscale quantification. Primary antibodies: AKT Polyclonal antibody (10176-2-AP, 1:8,000), Phospho-AKT (Ser473) Monoclonal antibody (66444-1-Ig, 1:8,000), and PPAR Gamma Polyclonal antibody (16643-1-AP, 1:2,500) were purchased from Proteintech; Anti-Glucose Transporter GLUT4 Rabbit pAb (P011101, 1:800) and β-actin Rabbit Monoclonal Antibody (LF212S, 1:8,000) were purchased from Epizyme Biomedical.

### 2.14 Fasting blood glucose measurement

During the experimental period, mice were fasted and blood was collected from the tail vein by puncture. Fasting blood glucose levels were measured using a Bayer portable glucometer.

### 2.15 H&E staining

Liver or adipose tissue samples were fixed with 4% paraformaldehyde, embedded in paraffin, and cut into 5 μm sections. The sections were dewaxed in xylene, rehydrated through a graded ethanol series, and subjected to hematoxylin and eosin (H&E) staining. Residual staining solution was rinsed off with running water. The sections were then dehydrated through a graded ethanol series, cleared in xylene, and mounted with neutral balsam. Histological structures were observed under a light microscope and photographed.

### 2.16 Statistical analysis

Data analysis was performed using SPSS 23.0 software. Continuous data are expressed as mean ± standard deviation (SD). Comparisons between two groups were conducted using the independent samples t-test. For comparisons among multiple groups, one-way analysis of variance (ANOVA) was used, followed by Tukey’s test for pairwise comparisons. A P value < 0.05 was considered statistically significant.

## 3 Results

### 3.1 Network pharmacology-based screening of the potential targets of active components from Angelica sinensis acting on obesity and insulin resistance

**Figure 1:**
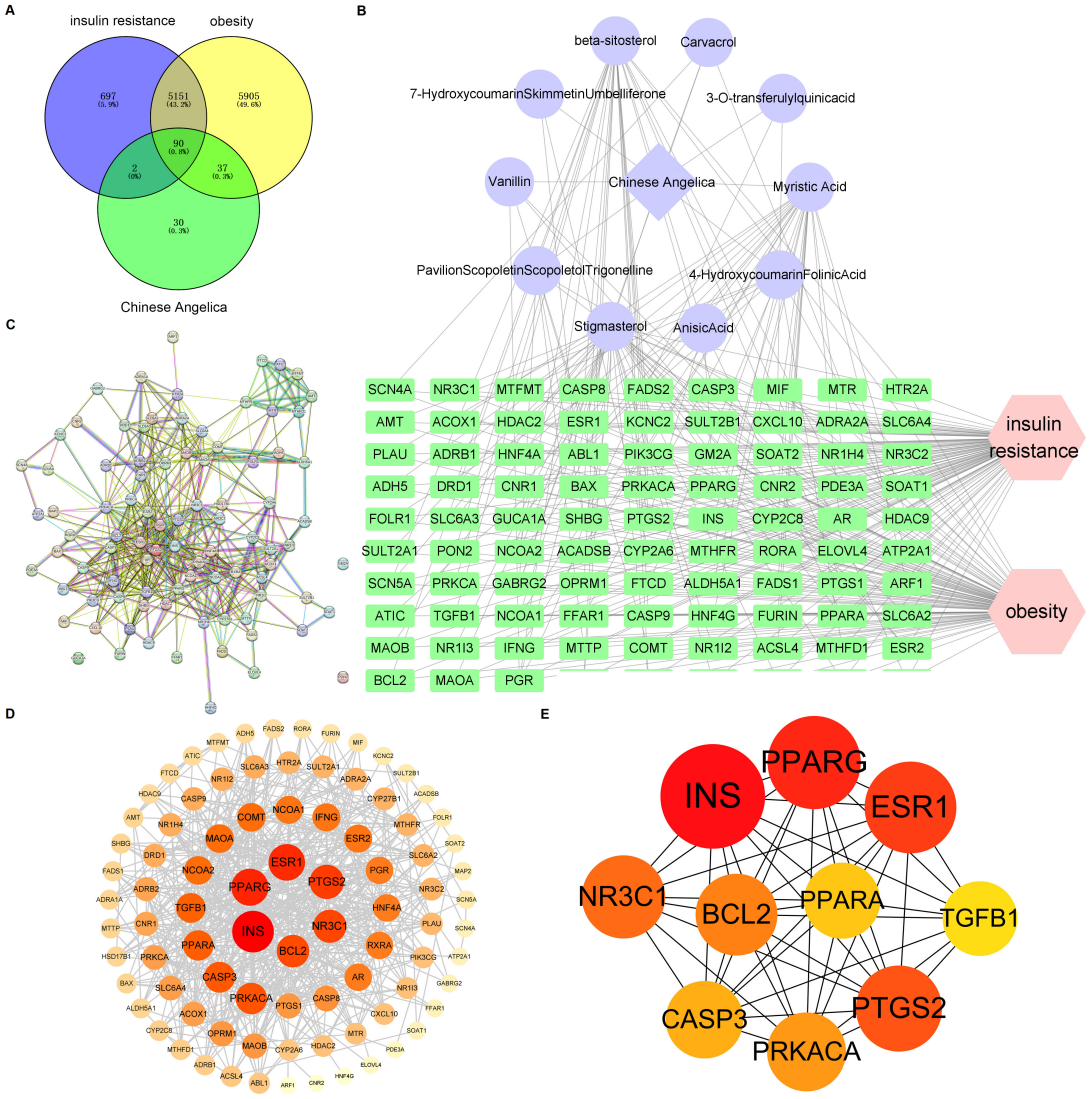
Target screening and network construction of the active components from Angelica sinensis acting on obesity and insulin resistance. **(A)** Venn diagram showing the target mapping of Angelica sinensis to obesity and insulin resistance, yielding 90 overlapping targets. **(B)** The “drug-active component-target” network diagram, containing a total of 103 nodes and 303 edges. **(C)** The protein-protein interaction network of the overlapping targets constructed using the STRING database. **(D)** The PPI network of the key targets obtained after topological analysis and screening by Cytoscape. **(E)** The PPI network of the core targets identified after further screening.

To systematically screen the potential active components and targets of Angelica sinensis involved in its intervention against obesity and insulin resistance, database retrieval and target mapping were first performed. A total of 90 overlapping targets between the drug and the diseases (obesity and insulin resistance) were obtained (**Figure 1A**). A “drug-active component-target” network was then constructed. As shown in **Figure 1B**, the network contained 103 nodes and 303 edges, visually demonstrating the multi-component and multi-target characteristics of Angelica sinensis. Among the active components, stigmasterol (Sti) showed relatively high connectivity within the network. To identify the key targets, a PPI network of the 90 overlapping targets was constructed based on the STRING database (**Figure 1C**), and topological analysis of the network was performed using Cytoscape software, yielding the core PPI network (**Figure 1D**). After further screening based on topological parameters such as degree, the top 10 targets ranked by degree were selected as the core targets (**Figure 1E**). Among these, PPARG, ESR1, INS, NR3C1, and BCL2 occupied central positions in the network, suggesting that they may play key roles in the mechanisms by which Angelica sinensis alleviates obesity-related insulin resistance.

### 3.2 GO and KEGG enrichment analyses and molecular docking validation

**Figure 2:**
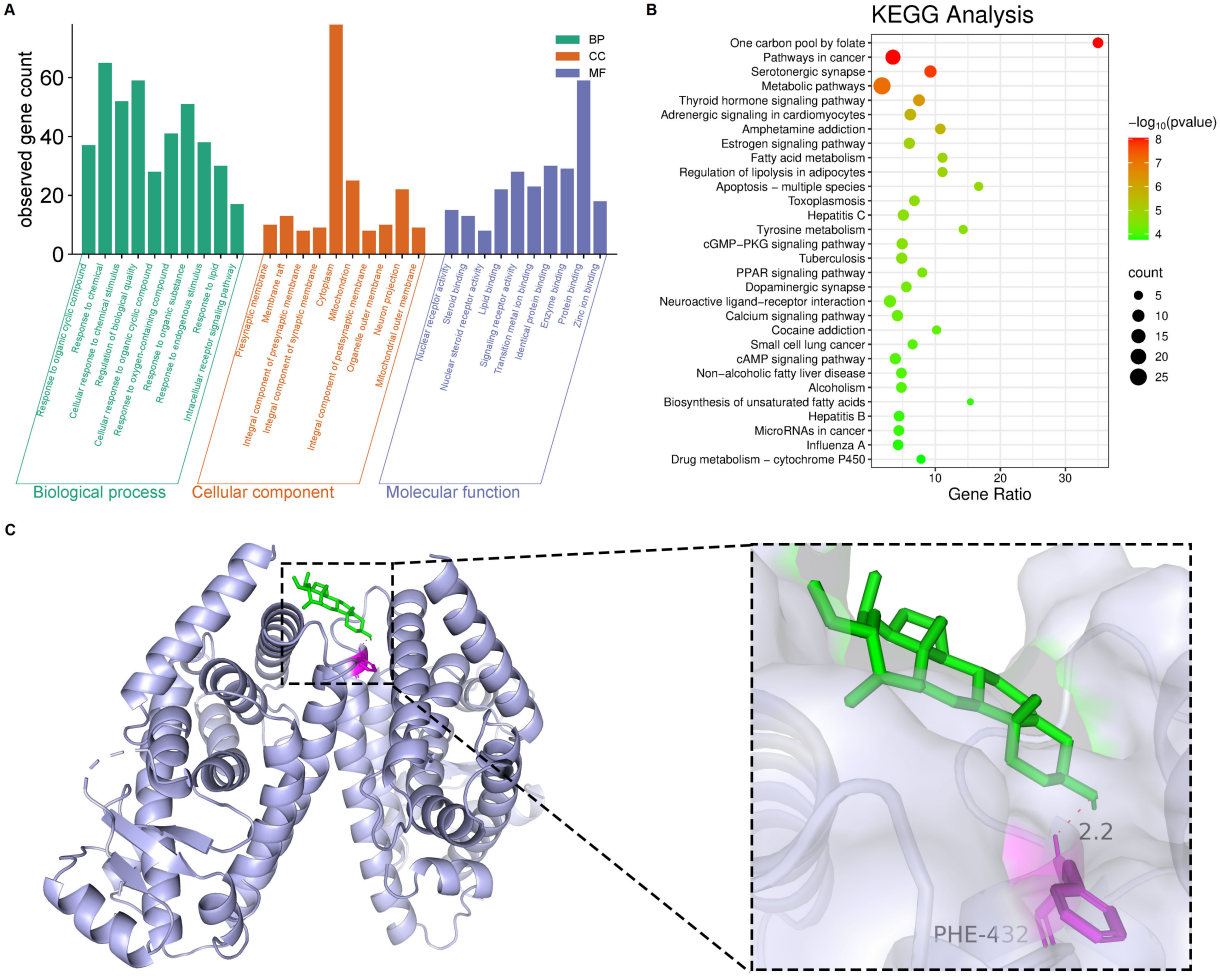
GO functional enrichment, KEGG pathway analysis, and molecular docking validation of the overlapping targets. **(A)** GO functional enrichment analysis, displaying the top 10 most significantly enriched terms in each of the following categories: BP, Biological Process; CC, Cellular Component; MF, Molecular Function. **(B)** Bubble plot of the top 30 enriched signaling pathways from KEGG pathway analysis. **(C)** Molecular docking model of stigmasterol bound to the PPARγ protein.

To investigate the biological functions and signaling pathways associated with the above targets, the 90 overlapping targets were subjected to GO and KEGG enrichment analyses. A total of 916 GO terms were enriched, and the top 10 terms in each category are presented in **Figure 2A**. The enriched terms in Biological Process (BP) were mainly involved in responses to organic cyclic compounds, cellular responses to chemical stimuli, responses to lipids, and intracellular receptor signaling pathways. In the Cellular Component (CC) category, the terms were primarily associated with membrane rafts, synaptic membranes, and mitochondria. The Molecular Function (MF) terms mainly involved nuclear receptor activity, steroid binding, and lipid binding. KEGG pathway enrichment analysis yielded 117 signaling pathways, and the top 30 pathways are displayed in **Figure 2B**. These pathways notably included the PPAR signaling pathway, fatty acid metabolism, regulation of lipolysis in adipocytes, non-alcoholic fatty liver disease, and the cAMP signaling pathway. Molecular docking was further performed to validate the binding capacity between the core component and key target. The results showed that Sti exhibited favorable binding affinity for the PPARγ protein (**Figure 2C**).

### 3.3 Effects of Sti on lipid accumulation in 3T3-L1 adipocytes

**Figure 3:**
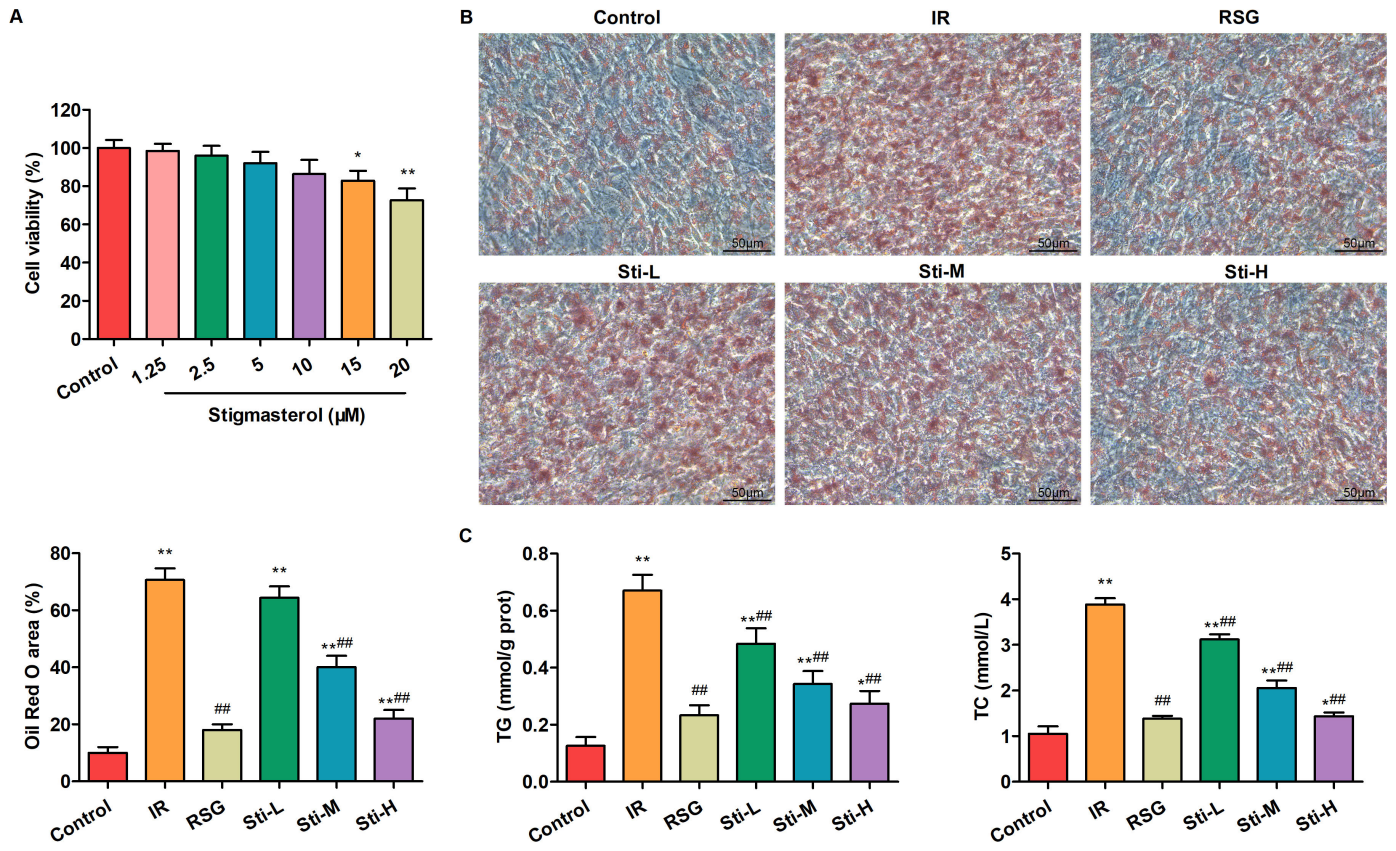
Effects of stigmasterol on 3T3-L1 adipocyte viability and lipid accumulation. **(A)** Cell viability of 3T3-L1 cells treated with different concentrations of stigmasterol as determined by the CCK-8 assay. **(B)** Oil Red O staining showing lipid droplet accumulation in each group (scale bar = 50 μm). **(C)** Quantitative analysis of intracellular TG and TC contents in each group. Data are presented as mean ± SD from three independent experiments (n = 3). ^*^p < 0.05, ^**^p < 0.01 vs. the Control group; ^#^p < 0.05, ^##^p < 0.01 vs. the IR group.

To determine the safe concentration range of Sti, the CCK-8 assay was used to evaluate the viability of 3T3-L1 cells treated with different concentrations of Sti. As shown in **Figure 3A**, Sti at 10 μM and below had no significant effect on cell viability. Therefore, 2.5, 5, and 10 μM were selected as the low, medium, and high doses (Sti-L, Sti-M, and Sti-H) for subsequent experiments. Oil Red O staining showed that, compared with the control group, intracellular lipid droplet accumulation was markedly increased in the IR model group. Compared with the IR group, all Sti-treated groups exhibited a concentration-dependent reduction in lipid droplet accumulation, and a similar reduction was observed in the rosiglitazone (RSG) positive control group (**Figure 3B**). Quantitative analysis of lipid content further confirmed that intracellular triglyceride (TG) and total cholesterol (TC) levels were significantly higher in the IR group than in the control group (p < 0.05), and both the Sti-L, Sti-M, Sti-H, and RSG groups showed significantly lower TG and TC levels than the IR group (p < 0.05) (**Figure 3C**).

### 3.4 Effects of Sti on ROS levels, antioxidant indicators, and PPARγ pathway protein expression in 3T3-L1 adipocytes

**Figure 4:**
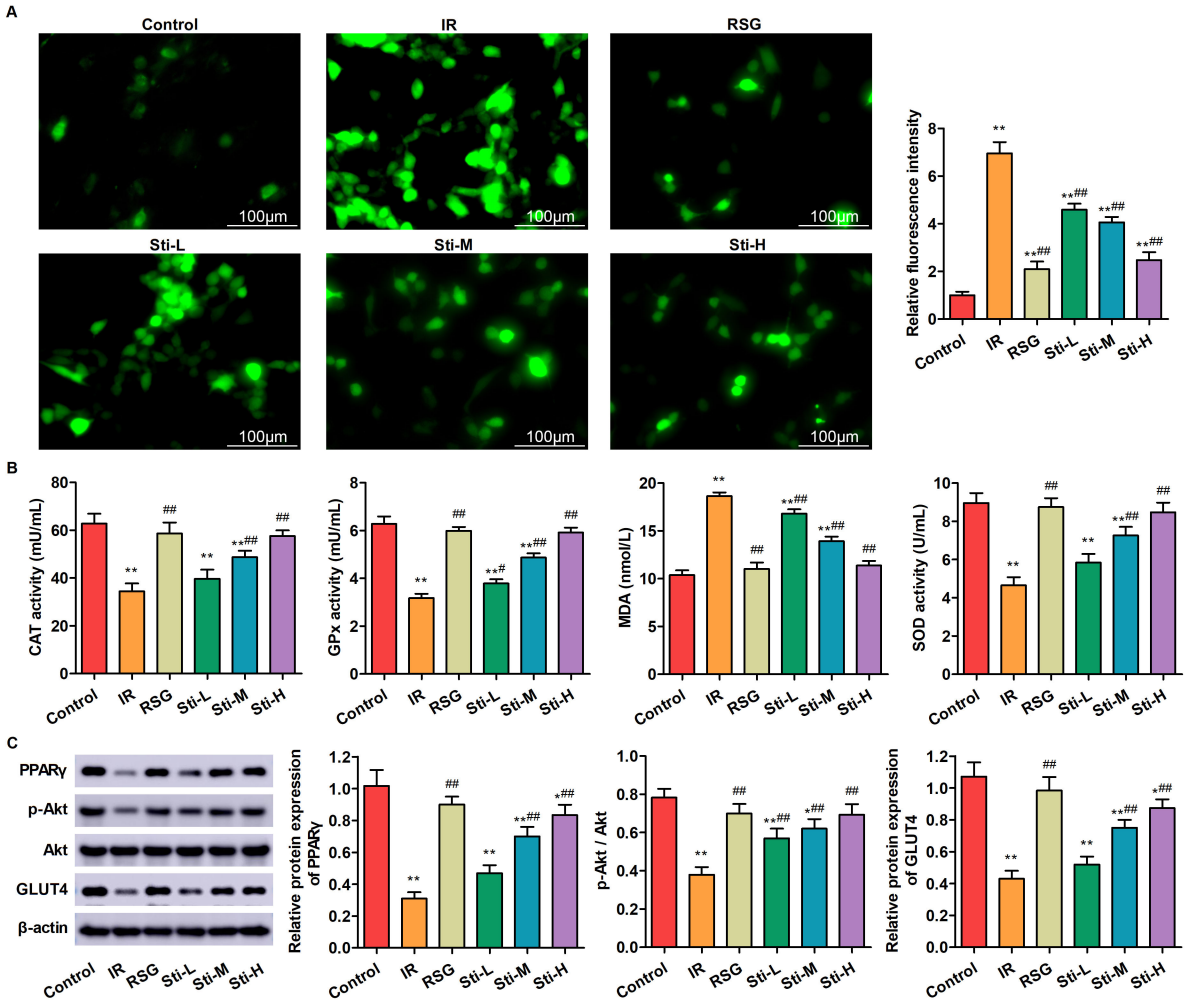
Effects of stigmasterol on oxidative stress and PPARγ pathway protein expression in 3T3-L1 adipocytes. **(A)** Detection of intracellular ROS levels by DCFH-DA probe and quantitative analysis of relative fluorescence intensity (scale bar = 100 μm). **(B)** Measurement of CAT, GPx, SOD activities and MDA content in each group. **(C)** Western blot analysis showing protein bands and quantitative analysis of PPARγ, p-Akt, Akt, and GLUT4 in each group. Data are presented as mean ± SD from three independent experiments (n = 3). ^*^p < 0.05, ^**^p < 0.01 vs. the Control group; ^#^p < 0.05, ^##^p < 0.01 vs. the IR group.

DCFH-DA probe detection showed that the ROS fluorescence intensity in the IR group was significantly higher than that in the Control group (p < 0.05), whereas the fluorescence intensity in all Sti-treated groups (Sti-L, Sti-M, Sti-H) and the RSG group was lower than that in the IR group (**Figure 4A**). For antioxidant indicators, the activities of CAT, GPx, and SOD in the IR group were significantly lower than those in the Control group (p < 0.05), while the MDA content was significantly higher (p < 0.05); these changes were reversed in all Sti-treated groups and the RSG group (**Figure 4B**). Western blot analysis showed that the protein expression levels of PPARγ, p-Akt/Akt, and GLUT4 in the IR group were decreased compared with the Control group (p < 0.05), whereas the expression of these proteins in all Sti-treated groups and the RSG group was increased compared with the IR group (p < 0.05, **Figure 4C**).

### 3.5 Effects of Sti on serum metabolic parameters in obese mice

**Figure 5:**
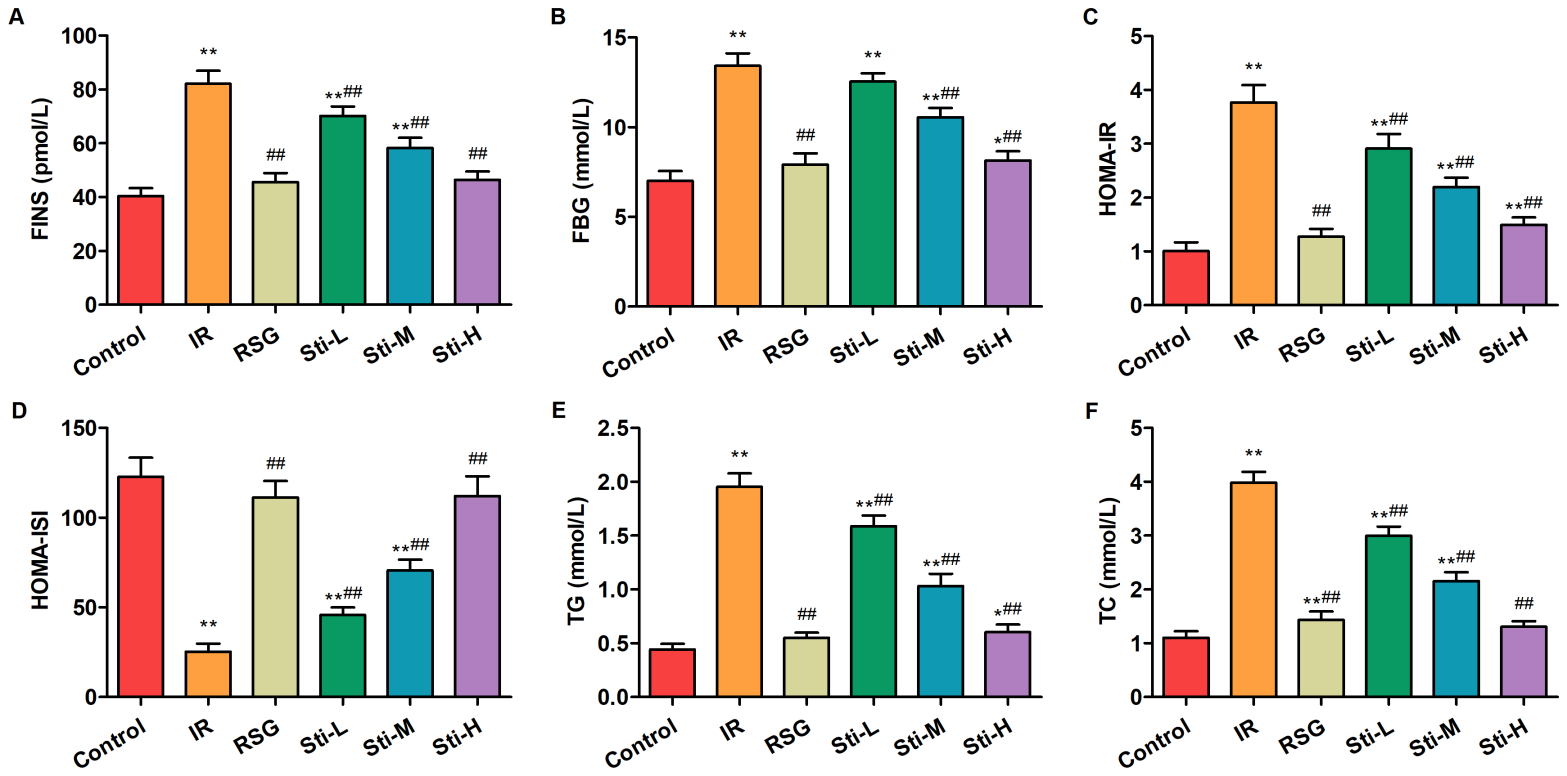
Effects of stigmasterol on serum metabolic parameters in obese mice. **(A)** Fasting insulin (FINS) levels in each group. **(B)** Fasting blood glucose (FBG) levels in each group. **(C)** HOMA-IR in each group. **(D)** HOMA-ISI in each group. **(E)** Serum TG levels in each group. **(F)** Serum TC levels in each group. Data are presented as mean ± SD (n = 6). ^*^p < 0.05, ^**^p < 0.01 vs. the Control group; ^#^p < 0.05, ^##^p < 0.01 vs. the IR group.

The results of serum analysis are shown in **Figure 5**. Compared with the Con group, the IR group had significantly higher levels of fasting insulin (FINS), fasting blood glucose (FBG), HOMA-IR, TG, and TC, and a significantly lower HOMA-ISI (p < 0.05). Compared with the IR group, the RSG group and all Sti-treated groups (Sti-L, Sti-M, Sti-H) showed varying degrees of reduction in FINS, FBG, HOMA-IR, TG, and TC levels, and varying degrees of increase in HOMA-ISI (p < 0.05).

### 3.6 Effects of Sti on the histopathology of adipose tissue and liver in obese mice

**Figure 6:**
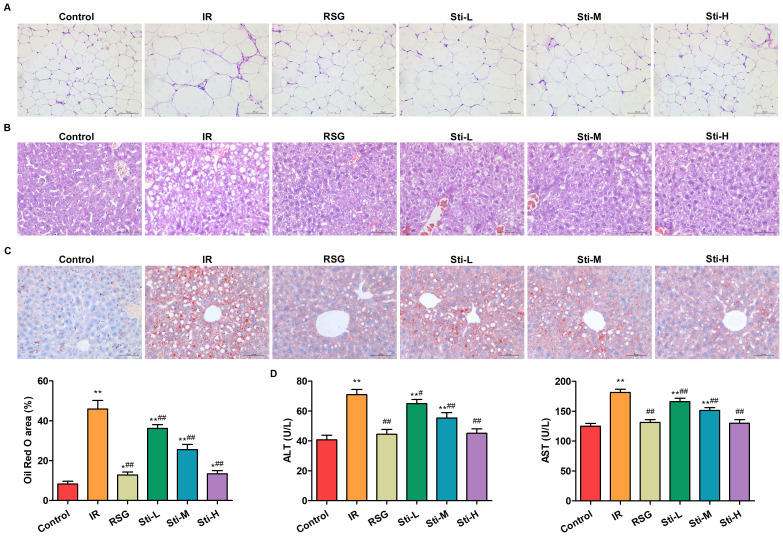
Effects of stigmasterol on the histopathology of adipose tissue and liver in obese mice. **(A)** Representative images of H&E-stained adipose tissue sections from each group (scale bar = 50 μm). **(B)** Representative images of H&E-stained liver tissue sections from each group (scale bar = 50 μm). **(C)** Representative images of Oil Red O-stained liver tissue sections from each group (scale bar = 50 μm). **(D)** Serum ALT and AST levels in each group. Data are presented as mean ± SD (n = 6). ^*^p < 0.05, ^**^p < 0.01 vs. the Control group; ^#^p < 0.05, ^##^p < 0.01 vs. the IR group.

The results of H&E staining of adipose tissue are shown in **Figure 6A**. Compared with the Control group, the adipocyte size in the IR group was markedly enlarged. Compared with the IR group, both the RSG group and all Sti-treated groups showed a reduction in adipocyte size to varying extents. H&E staining of liver tissue (**Figure 6B**) revealed that hepatocytes in the IR group were arranged in a disorganized manner, with numerous cytoplasmic lipid droplet vacuoles of varying sizes, when compared with the Control group. These lipid droplet vacuoles were reduced in the RSG group and all Sti-treated groups relative to the IR group. Oil Red O staining of liver tissue (**Figure 6C**) further showed that the area of red-stained lipid droplets was significantly increased in the IR group compared with the Control group (p < 0.05), and this area was reduced in the RSG group and all Sti-treated groups compared with the IR group. The results of serum ALT and AST measurements are presented in **Figure 6D**. The ALT and AST levels were significantly higher in the IR group than in the Control group (p < 0.05), and these levels were reduced to varying degrees in the RSG group and all Sti-treated groups compared with the IR group.

### 3.7 Effects of Sti on hepatic oxidative stress and PPARγ pathway protein expression in obese mice

**Figure 7:**
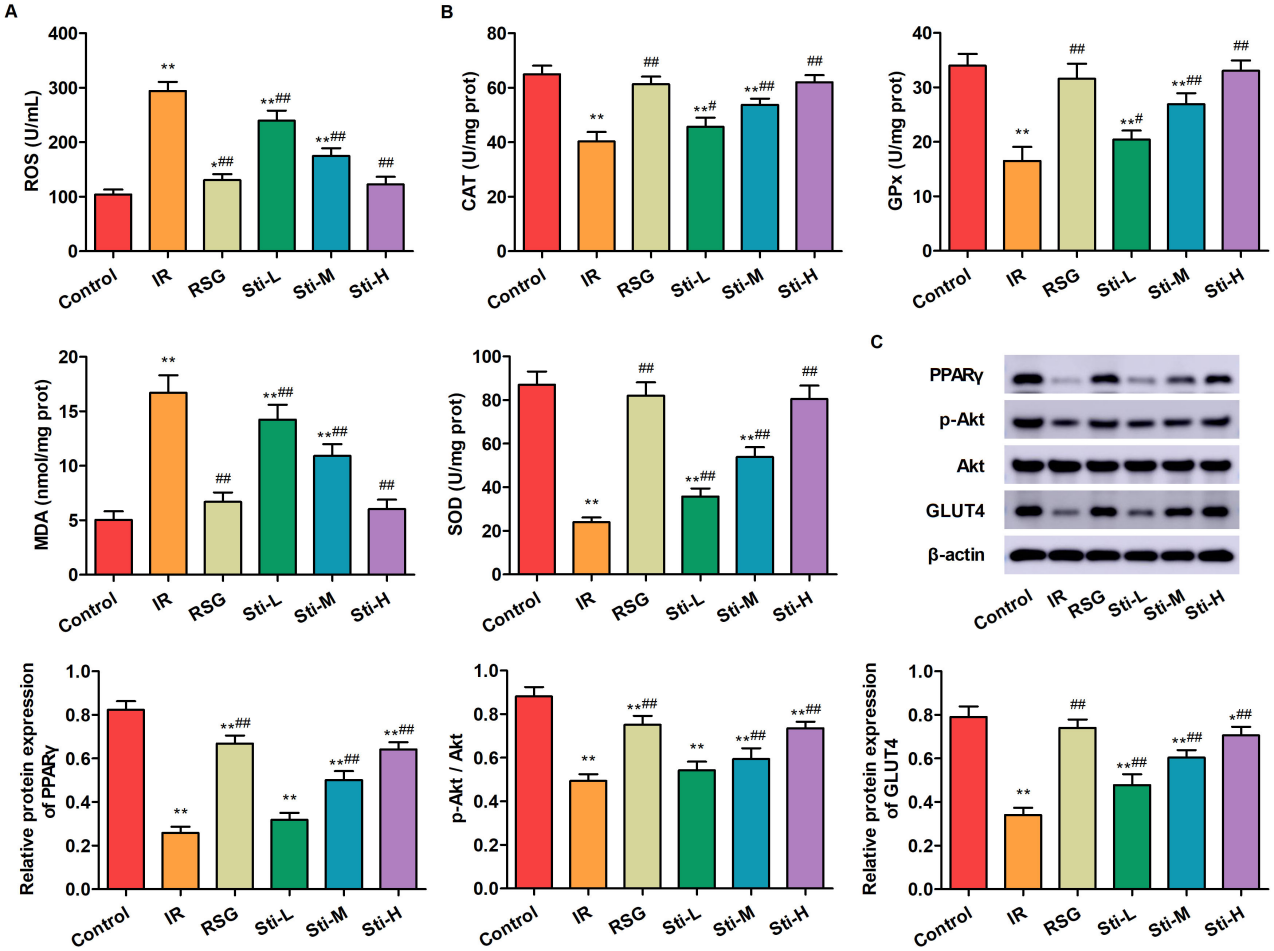
Effects of stigmasterol on hepatic oxidative stress and PPARγ pathway protein expression in obese mice. **(A)** Hepatic ROS levels in each group. **(B)** Hepatic CAT, GPx, SOD activities and MDA content in each group. **(C)** Western blot analysis showing protein bands and quantitative analysis of PPARγ, p-Akt, Akt, and GLUT4 in the liver of each group. Data are presented as mean ± SD (n = 6). ^*^p < 0.05, ^**^p < 0.01 vs. the Control group; ^#^p < 0.05, ^##^p < 0.01 vs. the IR group.

As shown in **Figure 7A**, the hepatic ROS level in the IR group was significantly higher than that in the Control group (p < 0.05). Compared with the IR group, the ROS levels in the Sti-L, Sti-M, Sti-H, and RSG groups were all significantly decreased (p < 0.05). The results of antioxidant assays (**Figure 7B**) showed that the activities of hepatic CAT, GPx, and SOD in the IR group were significantly lower than those in the Control group (p < 0.05), while the MDA content was significantly higher. Compared with the IR group, the activities of CAT, GPx, and SOD in the Sti-L, Sti-M, Sti-H, and RSG groups were significantly increased (p < 0.05), and the MDA content was significantly decreased (p < 0.05). Western blot analysis (**Figure 7C**) showed that the protein expression of PPARγ, the p-Akt/Akt ratio, and the protein expression of GLUT4 in the liver were significantly lower in the IR group than in the Control group (p < 0.05). Compared with the IR group, all of these indicators in the Sti-L, Sti-M, Sti-H, and RSG groups were significantly increased (p < 0.05).

## 4 Discussion

Based on the clues derived from network pharmacology screening and molecular docking, the present study systematically examined the effects of stigmasterol (Sti) on metabolic phenotypes, oxidative stress, and the PPARγ pathway in an insulin-resistant 3T3-L1 adipocyte model and in high-fat diet-induced obese mice. The results demonstrated that Sti ameliorated obesity-related glycolipid metabolic disorders and insulin resistance both *in vitro* and *in vivo*. Given that previous studies on the metabolic effects of Sti have largely focused on its cholesterol-lowering and anti-inflammatory properties (19, 20), and its effects in insulin resistance models have not been systematically reported, the present study fills this gap.

Cell experiments showed that Sti intervention reduced intracellular TG and TC accumulation in insulin-resistant adipocytes. Animal experiments further confirmed that Sti decreased the serum levels of fasting insulin, fasting blood glucose, HOMA-IR, and blood lipids, increased HOMA-ISI, reduced adipocyte size, alleviated hepatic lipid deposition, and lowered ALT and AST levels in obese mice. These effects were consistent with the trend observed for rosiglitazone. As a full PPARγ agonist, rosiglitazone improves insulin resistance through mechanisms involving the regulation of lipid metabolism and adiponectin secretion (21, 22); however, whether the lipid metabolism-improving effect of Sti also involves PPARγ-mediated modulation of adiponectin secretion remains to be clarified in future studies. Furthermore, hepatic ectopic lipid deposition in obesity is an important factor that aggravates insulin resistance (23). In this study, Sti intervention reduced the degree of hepatic steatosis, accompanied by an improvement in systemic insulin sensitivity (reflected by a reduced HOMA-IR), suggesting that Sti exerts dual beneficial effects on hepatic lipid metabolism and insulin resistance.

Oxidative stress plays a key driving role in the pathogenesis of obesity-related insulin resistance. In obesity, mitochondrial dysfunction in adipose tissue leads to excessive production of ROS (24). ROS can activate JNK, which on one hand upregulates JUN to suppress insulin signaling, and on the other hand promotes serine phosphorylation of IRS-1, leading to AKT inactivation and thereby inducing insulin resistance (25, 26). In addition, oxidative stress can induce endoplasmic reticulum stress, further aggravating insulin resistance through the PERK/eIF2α and IRE1/JNK pathways (27). In this study, we observed significantly increased ROS and MDA levels and significantly decreased CAT, GPx, and SOD activities in the IR model cells, which is consistent with the oxidative-antioxidative imbalance reported in the literature (28). These indicators were markedly restored after Sti intervention. The *in vitro* free radical scavenging activity of Sti has been previously reported (29). The present study extends its antioxidant effects to the pathological context of insulin resistance and establishes a link between these effects and metabolic improvement.

Regarding the molecular mechanism by which Sti exerts the above effects, this study focused on the PPARγ pathway. PPARγ is highly expressed in adipose tissue, and its activation not only regulates lipid metabolism but has also been confirmed in recent years to directly act on PPRE elements in the promoter regions of antioxidant enzyme genes such as CAT and SOD, thereby initiating antioxidant gene transcription (30, 31). At the level of insulin signaling, PPARγ activation can promote GLUT4 translocation to the cell membrane through the PI3K/Akt pathway (32), and PTEN downregulation may also contribute to the maintenance of Akt phosphorylation (33). Taken together, our results showed that Sti intervention upregulated PPARγ protein expression, accompanied by the restoration of antioxidant enzyme activities, a reduction in ROS levels, and the recovery of Akt phosphorylation and GLUT4 expression. These changes suggest that Sti may activate PPARγ to, on the one hand, enhance antioxidant defense and attenuate oxidative stress, and on the other hand, restore the function of the Akt/GLUT4 pathway; both mechanisms work in concert to ameliorate insulin resistance. This pattern is consistent with the characteristics of certain natural product-derived PPARγ partial agonists (34, 35). The specific molecular mechanism by which Sti upregulates PPARγ protein expression still requires further elucidation in future studies.

In conclusion, this study is the first to demonstrate, both *in vitro* and *in vivo*, that stigmasterol can ameliorate obesity-related insulin resistance, and its mechanism is associated with activation of the PPARγ pathway and attenuation of oxidative stress. This finding provides an experimental basis for developing Sti as a safe metabolic intervention targeting the PPARγ/oxidative stress axis, and also offers a new direction for identifying insulin sensitizers from phytosterols.

## Data Availability

The original contributions presented in the study are included in the article/supplementary material. Further inquiries can be directed to the corresponding author.
